# Efficacy of chimeric antigen receptor T cell therapy and autologous stem cell transplant in relapsed or refractory diffuse large B-cell lymphoma: A systematic review

**DOI:** 10.3389/fimmu.2022.1041177

**Published:** 2023-01-17

**Authors:** Linyan Tian, Cheng Li, Juan Sun, Yixin Zhai, Jinhuan Wang, Su Liu, Yanan Jiang, Wenqi Wu, Donghui Xing, Yangyang Lv, Jing Guo, Hong Xu, Huimeng Sun, Yuhang Li, Lanfang Li, Zhigang Zhao

**Affiliations:** ^1^ Department of Hematology, Tianjin Medical University Cancer Institute and Hospital, National Clinical Research Center for Cancer, Key Laboratory of Cancer Prevention and Therapy, Tianjin’s Clinical Research Center for Cancer, Tianjin, China; ^2^ Clinical Testing Center, Chinese Academy of Medical Sciences Blood Disease Hospital, Chinese Academy of Medical Sciences Institute of Hematology, State Key Laboratory of Experimental Hematology, National Clinical Medical Center for Blood Disease, Tianjin, China; ^3^ Department of Oncology, Second Hospital of Tianjin Medical University, Institute of Urology, Tianjin, China; ^4^ Department of Lymphoma, Tianjin Medical University Cancer Institute and Hospital, National Clinical Research Center of Cancer, Key Laboratory of Cancer Prevention and Therapy, Tianjin’s Clinical Research Center for Cancer, The Sino‐US Center for Lymphoma and Leukemia Research, Tianjin, China; ^5^ Department of Medical Oncology, Tianjin First Central Hospital, School of Medicine, Nankai University, Tianjin, China

**Keywords:** R/R DLBCL, CAR-T cell, auto-HSCT, survival, meta-analysis

## Abstract

**Background:**

We aimed to compare the efficacy of chimeric antigen receptor T (CAR-T) cell therapy with that of autologous stem cell transplantation (auto-HSCT) in relapsed/refractory diffuse large B cell lymphoma (R/R DLBCL).

**Research design and methods:**

We searched eligible publications up to January 31st, 2022, in PubMed, Cochrane Library, Springer, and Scopus. A total of 16 publications with 3484 patients were independently evaluated and analyzed using STATA SE software.

**Results:**

Patients who underwent CAR-T cell therapy showed a better overall response rate (ORR) and partial response (PR) than those treated with auto-HSCT (CAR-T vs. auto-HSCT, ORR: 80% vs. 73%, HR:0.90,95%CI:0.76-1.07,*P* = 0.001; PR: 20% vs. 14%, HR:0.65,95%CI:0.62-0.68,*P* = 0.034). No significant difference was observed in 6-month overall survival (OS) (CAR-T vs. auto-HSCT, six-month OS: 81% vs. 84%, HR:1.23,95%CI:0.63-2.38, *P* = 0.299), while auto-HSCT showed a favorable 1 and 2-year OS (CAR-T vs. auto-HSCT, one-year OS: 64% vs. 73%, HR:2.42,95%CI:2.27-2.79, P < 0.001; two-year OS: 54% vs. 68%, HR:1.81,95%CI:1.78-1.97, P < 0.001). Auto-HSCT also had advantages in progression-free survival (PFS) (CAR-T vs. auto-HSCT, six-month PFS: 53% vs. 76%, HR:2.81,95%CI:2.53-3.11,*P* < 0.001; one-year PFS: 46% vs. 61%, HR:1.84,95%CI:1.72-1.97,*P* < 0.001; two-year PFS: 42% vs. 54%, HR:1.62,95%CI:1.53-1.71, *P* < 0.001). Subgroup analysis by age, prior lines of therapy, and ECOG scores was performed to compare the efficacy of both treatment modalities.

**Conclusion:**

Although CAR-T cell therapy showed a beneficial ORR, auto-HSCT exhibited a better long-term treatment superiority in R/R DLBCL patients. Survival outcomes were consistent across different subgroups.

## 1 Introduction

Diffuse large B-cell lymphoma (DLBCL) is the most common subtype of lymphoma in adults worldwide. It accounts for 30–40% of newly diagnosed non-Hodgkin lymphomas (NHLs) annually, with an increasing incidence (1, 2). Although combination chemotherapy with rituximab plus cyclophosphamide, doxorubicin, vincristine, and prednisone (CHOP) serves as the backbone of treatment, up to 40% of patients experience treatment failure or inevitable relapse (3, 4). Patients with relapsed/refractory (R/R) DLBCL have particularly poor outcomes (5, 6).

High-dose chemotherapy with autologous stem cell transplantation (auto-HSCT) is a promising therapeutic avenue and standard of care for patients with R/R DLBCL (7). In 1995, a randomized trial of auto-HSCT (PARMA) first demonstrated the benefit of transplantation in patients with R/R DLBCL (8). In recent years, a prospective, multicenter phase II clinical trial reported that the estimated overall survival (OS) and progression-free survival (PFS) rates were 63% and 61%, respectively, three years after auto-HSCT, with a median follow-up of 31.0 months ((EudraCT) N. 2007-003198-22) (9). In addition, auto-HSCT has led to satisfactory outcomes in older adults. The European Blood and Marrow Transplantation (EBMT) registry demonstrated a three-year OS of 60% after auto-HSCT in older patients (10). The Canadian Cancer Trials Group(CCTG) LY.12 study indicated that the older group gained similar benefits from auto-HSCT compared to the younger group, with an acceptable safety profile (NCT00078949) (11).

As a newly developed cell immunotherapy, chimeric antigen receptor T (CAR-T) cell therapy continues to be a suitable treatment option and has shown remarkable achievements in B-cell malignancies (12, 13). In TRANSCEND NHL 001, CAR-T cell therapy resulted in a considerable objective response rate (ORR), with a low incidence of serious cytokine release syndrome and neurological events (NCT02631044) (14). An open-label, multicenter, international phase II study reported an overall response rate (ORR) of 52% and complete response (CR) rate of 40% after infusion of CAR-T products (NCT02445248) (15). In a phase I-II ZUMA-1 study with CAR-T cell therapy, 82% of patients had an objective response and 58% achieved CR (NCT02348216) (16). In ZUMA-7, the median event-free survival (EFS) in the CAR-T group was 8.3 months and the estimated event-free survival at 24.0 months was 41% (95% CI, 33%–48%) (NCT03391466) (17). Different anti-CD19 CAR-T cell infusion products have been used in patients with R/R DLBCL. Axicabtagene ciloleucel (axi-cel) was the first product approved in 2017, followed by tisagenlecleucel (tisa-cel) in 2018 and lisocabtagene maraleucel (liso-cel) in 2021 (18–20). CAR-T cell therapy exhibited a remarkable efficacy and acceptable safety profile. It has been successfully used for patients with R/R DLBCL and is rapidly becoming the standard of care (21).

According to the Center for International Blood & Marrow Transplant Research(CIBMTR) study, a decreasing number of patients in America are choosing auto-HSCT after salvage treatment in the CAR-T era (22, 23). The application of auto-HSCT or CAR-T cell therapy has been considered in patients with R/R DLBCL (24). According to a comparative study, CAR-T cell therapy exhibited a superior clinical outcome over auto-HSCT for efficacy (NCT03196830) (CAR-T vs. auto-HSCT, one-year OS 74.4% vs. 44.5%, *P* = 0.044) (25). In contrast, improved survival was reported in the auto-HSCT group vs. the CAR-T group in a large retrospective study (CAR-T vs. auto-HSCT, two-year OS 69% vs. 47%, *P* = 0.004) (26). Similarly, according to a BELINDA clinical trial, no apparent survival benefit was observed in CAR-T cell therapy (NCT03570892, median EFS 3.3 months vs. 3.0 months, *P* = 0.61). So far, no universal agreement in the option between auto-HSCT or CAR-T cell therapy in R/R DLBCL has been reached. Therefore, our study aimed to compare the efficacy of auto-HSCT with CAR-T cell therapy for R/R DLBCL.

## 2 Methods

### 2.1 Literature search

**Figure 1:**
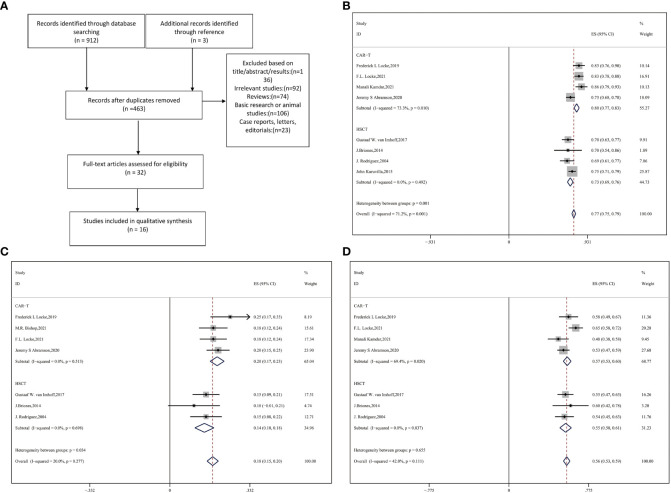
**(A)** Flow diagram of selecting eligible studies Forest plot of **(B)** ORR **(C)** PR **(D)** CR between CAR-T and auto-HSCT groups.

The search strategy developed according to the Preferred Reporting Items for Systematic Reviews and Meta-analyses (PRISMA) reporting guidelines is shown in **Figure 1A**. Eligible studies, with the latest update on January 31, 2022, were searched for using the PubMed, Cochrane Library, Springer, and Scopus databases. We combined the search terms *DLBCL/diffuse large B-cell lymphoma*, *CAR-T/chimeric antigen receptor T*, *and ASCT/autologous stem cell transplantation/HCT* to identify potential studies without language restrictions.

### 2.2 Inclusion and exclusion criteria

Studies were included if they met the following criteria (1): prospective clinical trials or retrospective studies; (2) patients with R/R DLBCL (diagnoses were rendered according to the WHO 2016 classification); (3) patients treated with CAR-T cell therapy or auto-HSCT; and (4) studies that reported at least one of the following outcome measures: ORR, CR, partial response (PR), OS, and PFS.

The exclusion criteria were as follows: (1) studies that were reviews, viewpoints, perspectives, or correspondences; (2) lack of effective data on the outcomes mentioned above; (3) basic research or animal studies; and (4) duplicate publications.

### 2.3 Study qualitative assessment and bias risk

As both randomized clinical trials (RCT) and nonrandomized clinical trials were involved, we adopted the Methodological Index for Non-randomized Studies (MINORS) to assess the quality of the included studies. Each item was scored as 0 (not reported), 1 (reported but inadequate), or 2 (reported and adequate) (27). We conducted the Begg’s and Egger’s tests to evaluate publication bias.

### 2.4 Data collection

The following information was collected and extracted by different authors independently: first author, publication year, study design, number of enrolled patients and those who received treatment, median age, and efficacy outcomes (ORR, CR, PR, OS, and PFS). The response rate was evaluated at 6-8 weeks after CAR-T cell or auto-HSCT therapy. The ORR was defined as the combined percentage of patients who had a complete or PR according to the Lugano classification. OS was defined as the time from treatment to death from any cause. PFS was defined as the time from treatment to the first date of disease progression (28). Conventional chemotherapy regimens included rituximab (R-ICE, R-GDP), and lymphodepleting chemotherapy was administered before CAR-T cell therapy and auto-HSCT. Disputes were addressed by a third reviewer or through group discussion.

### 2.5 Statistical analysis

We used STATA SE software (version 12.0; StataCorp, College Station, TX, USA) to analyze the therapeutic efficacy and safety. Additionally, the I² statistic was used to test for heterogeneity. A fixed-effects model was used to calculate the pooled effects when I² < 50%. Otherwise, a random effects model was adopted. We performed stratified analysis and explored the sources of heterogeneity. Statistical significance was set at *P* < 0.05.

## 3 Results

The flowchart in **Figure 1A** depicts the search process. A total of 912 studies were accessible from the different databases used, of which 452 duplicates were excluded. At the same time, another three studies available in the references were considered relevant. Based on the exclusion criteria, 16 publications with 3484 total patients enrolled were ultimately included (9, 14–17, 26, 29–38). Eventually, 1067 patients with R/R DLBCL who received anti-CD19 CAR-T cells and 2417 patients who underwent auto-HSCT were evaluated.

As illustrated in **Table 1**, the included studies were published between 2004 and 2021. Eligible patients had R/R DLBCL (defined as a relapse or progressive or stable disease as the best response to the most recent therapy). The median age of the enrolled patients ranged from 40 to 64 years in both the groups. Most patients had stage III or IV disease and had received several previous lines of systemic therapy (**Table 1**). The median follow-up of the included studies ranged from 6.2 months to 55.2 months. The primary outcome was the response rate (ORR, CR, and PR), and the secondary outcomes were OS and PFS.

**Table 1:** Characteristics of included studies.

Treatment	Name,Year	Study Design	N	Median age	Median Number of Prior Therapy	Outcomes	MINORS
CAR-T	Stephen J. Schuster,2019 (15)	prospective	111	56	NR	PFS	16
CAR-T	Frederick L Locke,2019 (16)	prospective	108	58	3	ORR CR PR OS PFS	16
CAR-T	F.L. Locke,2021 (17)	RCT	180	59	1	ORR CR PR OS PFS	24
CAR-T	Manali Kamdar,2021 (30)	RCT	92	60	1	ORR CR PR	24
CAR-T	Mazyar Shadman,2022A (26)	retrospective	145	60	3	OS PFS	22
CAR-T	Jeremy S Abramson,2020 (14)	prospective	269	63	3	ORR CR OS PFS	16
CAR-T	M.R. Bishop, 2021 (29)	RCT	162	60	1	PR	16
HSCT	Christopher R. Flowers,2017 (31)	prospective	63	51	NR	OS PFS	16
HSCT	Daria Gaut,2019 (32)	retrospective	111	57	NR	OS PFS	14
HSCT	John Kuruvilla,2015 (33)	prospective	429	56	1	OS PFS	16
HSCT	Gustaaf W. van Imhoff,2017 (34)	prospective	447	57	NR	ORR CR PR PFS	16
HSCT	Julie M. Vose,2013 (35)	RCT	224	58	2	OS PFS	24
HSCT	Mazyar Shadman,2022B (26)	retrospective	266	58	2	OS PFS	22
HSCT	J.Briones,2014 (9)	prospective	30	53	3	ORR CR PR OS PFS	16
HSCT	J. Rodriguez,2004 (36)	retrospective	114	40	NR	ORR CR PR OS	14
HSCT	Dai Chihara,2014 (37)	retrospective	484	64	3	OS	14
HSCT	Nirav N. Shah,2020 (38)	retrospective	249	59	NR	OS PFS	14

Overall, the included studies were reliable, according to the MINORS scale. The detailed scores are listed in **Table 1**. Begg’s and Egger’s tests were performed to evaluate the bias more precisely. Despite the limited number of studies involved in assessing publication bias, there was no evident publication bias for response rate(Begg’s test *P* =0.902; Egger’s test *P* =0.803), OS(Begg’s test *P* =0.276; Egger’s test *P* =0.210), and PFS(Begg’s test *P* =1.000; Egger’s test *P* =0.513) in our evaluation (**Figure S1**).

### 3.1 Response rate

Eight studies with 1402 patients in total reported ORR and response extent (CR and PR) (9, 16, 17, 25, 29, 30, 34, 36). Compared with auto-HSCT, CAR-T cell therapy performed significantly better in terms of ORR and PR (CAR-T vs. auto-HSCT, ORR: 80% vs. 73%, HR:0.90,95%CI:0.76-1.07,*P* = 0.001; PR: 20% vs. 14%, HR:0.65,95%CI:0.62-0.68,*P* = 0.034) (**Figures 1B, C**). However, no significant difference in CR was observed between the two groups (CAR-T vs. auto-HSCT, CR: 57% vs. 55%, HR:0.92,95%CI:0.842-0.986, *P* = 0.655) (**Figure 1D**).

### 3.2 OS

**Figure 2:**
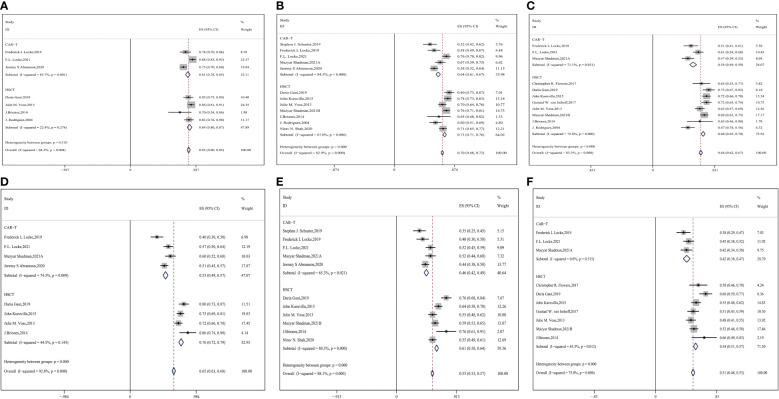
Forest plot of OS between CAR-T and auto-HSCT groups **(A)** 6-month OS **(B)** 1-year OS **(C)** 2-year OS Forest plot of PFS between CAR-T and auto-HSCT groups **(D)** 6-month PFS **(E)** 1-year PFS **(F)** 2-year PFS.

Twelve studies involving 2672 patients were involved in the appraisal of short- and long-term OS (9, 14, 16, 17, 26, 31–33, 35–38). We pooled all of them and analyzed the OS. The results are shown in **Figures 2A-C** (CAR-T vs. auto-HSCT, six-month OS: 81% vs. 84%, HR:1.23,95%CI:0.63-2.38, *P*=0.310 (**Figure 2A**); one-year OS: 64% vs. 73%, HR:2.42,95%CI:2.27-2.79, *P* < 0.001; two-year OS: 54% vs. 68%, HR:1.81,95%CI:1.78-1.97, *P* < 0.001). This finding indicates that even though no significant differences in short-term OS were observed between the CAR-T and auto-HSCT groups, patients who underwent auto-HSCT benefited from long-term OS.

### 3.3 PFS

Twelve studies with 2632 patients were assessed for PFS, and **Figure 2** shows the meta-analysis forest diagram of PFS (9, 14–17, 26, 31–35, 38). Auto-HSCT showed benefits at six months, one year, and two years of PFS compared with the CAR-T cell group (CAR-T vs. auto-HSCT, six-month PFS: 53% vs. 76%, HR:2.81,95%CI:2.53-3.11,*P* < 0.001; one-year PFS: 46% vs. 61%, HR:1.84,95%CI:1.72-1.97,*P* < 0.001; two-year PFS: 42% vs. 54%, HR:1.62,95%CI:1.53-1.71, *P* < 0.001) (**Figures 2D–F**). This finding indicates that patients who underwent auto-HSCT were more likely to have a better PFS than those who underwent CAR-T cell therapy.

### 3.4 Subgroup analysis

#### 3.4.1 Subgroup analysis by age

**Figure 3:**
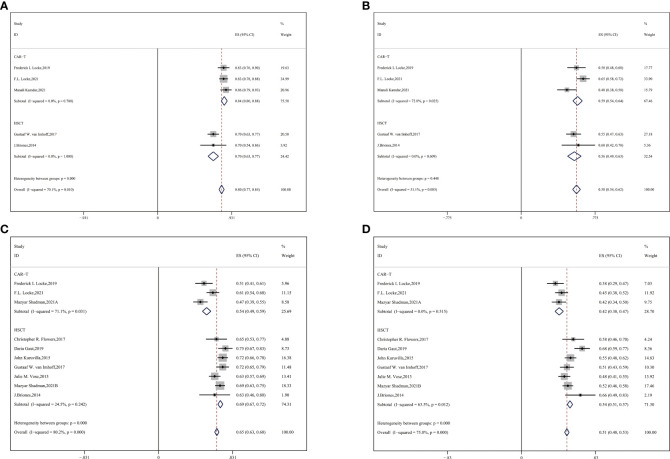
Subgroup analyses by age between CAR-T and auto-HSCT groups **(A)** ORR **(B)** CR **(C)** 2-year OS **(D)** 2-year PFS.

Subgroup analysis by age showed that among the mean age of 50–60 groups, the ORR was higher in CAR-T cell groups and CR was approximately equivalent to each other (CAR-T vs. auto-HSCT, ORR: 84% vs. 70%, HR:0.45,95%CI:0.39-0.51,*P* < 0.001; CR: 59% vs. 56%, HR:0.88,95%CI:0.82-0.94,*P* = 0.448) (**Figures 3A, B**). Auto-HSCT showed an advantage in OS and PFS over the CAR-T cell group (CAR-T vs. auto-HSCT, two-year OS: 54% vs. 69%, HR:1.90, 95%CI:1.74-2.08, *P* < 0.001; two-year PFS: 42% vs. 54%, HR:1.62,95%CI:1.53-1.72, *P* < 0.001) (**Figures 3C, D**).

#### 3.4.2 Subgroup analysis by previous therapeutic lines

**Figure 4:**
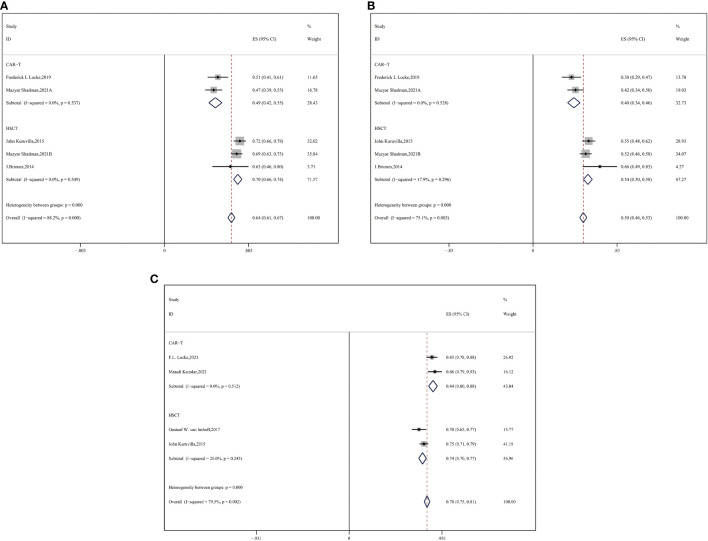
Subgroup analysis by previous therapeutic lines between CAR-T and auto-HSCT groups **(A)** 2-year OS **(B)** 2-year PFS **(C)** ORR.

Most patients with R/R DLBCL had previously undergone a couple of systematic therapeutic lines, so we performed a subgroup analysis and observed an advantage of two-year OS and PFS in the auto-HSCT group compared with the CAR-T group. For median previous therapies of no more than three (≤3) lines, more enrolled patients with R/R DLBCL gained benefits of survival in the auto-HSCT vs. CAR-T cell group (CAR-T vs. auto-HSCT, two-year OS: 49% vs. 70%, HR:2.43,95%CI:2.03-2.86, *P* < 0.001; two-year PFS: 40% vs. 54%, HR:1.76,95%CI:1.67-1.86, *P* < 0.001) (**Figures 4A, B**).

We compared the ORR between the subgroups of auto-HSCT and CAR-T cell therapy as second-line treatment. CAR-T cell therapy resulted in a superior ORR for the auto-HSCT group (CAR-T vs. auto-HSCT, ORR: 84% vs. 74%, HR:0.54,95%CI:0.47-0.61, *P* < 0.001) (**Figure 4C**).

#### 3.4.3 Subgroup analysis by ECOG scores

**Figure 5:**
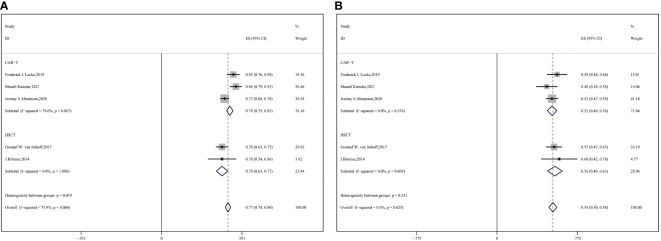
Subgroup analysis by ECOG scores between CAR-T and auto-HSCT groups **(A)** ORR **(B)** CR.

Subsequently, we performed subgroup analysis of patients with ECOG scores ranging from 0–2. In this subgroup analysis, a higher ORR was observed in the CAR-T cell group (CAR-T vs. auto-HSCT, ORR: 79% vs. 70%, HR:0.62,95%CI:0.54-0.70, *P* = 0.019) (**Figure 5A**). No significant difference was observed in CR between the two groups (53% vs. 56%, HR:1.13,95%CI:1.06-1.19, *P* = 0.531) (**Figure 5B**). In our study, CAR-T cell therapy showed an improved ORR and a similar PR compared to the auto-HSCT group.

Survival outcomes were consistent across the subgroups described above. CAR-T cell therapy showed a favorable response rate, whereas auto-HSCT exhibited promising long-term survival.

## 4 Discussion

DLBCLs are a heterogeneous group of aggressive B-cell neoplasms with different clinical prognoses (39, 40). Patients with R/R DLBCL have poor prognosis despite a variety of salvage therapies. The recent NCCN guidelines recommend high-dose chemotherapy supported by auto-HSCT for patients with R/R DLBCL eligible for transplant (41). The advent of CAR-T cell therapy has reduced the number of patients undergoing auto-HSCT. Currently, CAR-T cell therapy and auto-HSCT are recommended for patients with R/R DLBCL. We compared the efficacy of CAR-T cell therapy and auto-HSCT in patients with R/R DLBCL. In our study, although CAR-T cell therapy was associated with a higher initial remission rate, auto-HSCT showed superior long-term survival.

A comparison between pivotal clinical trials and CAR-T cell products indicated an ORR ranging from 59% to 82% in patients with R/R DLBCL (42), which was better than that of auto-HSCT, as reported previously. The ORR outcomes in our study are consistent with previous observations that CAR-T cell therapy results in a better ORR. As previously confirmed in ZUMA-1, a high CAR-T cell peak concentration in the first 28 days after infusion contributed to a remarkable ORR in the CAR-T cell group (16).

A retrospective study of the EBMT center reported a five-year OS of 63% and five-year disease free survival of 48% after auto-HSCT (43). Since CAR-T cell therapy was developed in recent years and has been recommended as a novel agent for R/R DLBCL, it has been shown to achieve satisfactory efficacy and has attracted increasing attention in this field (44). In 2021, two-year survival outcomes of randomized phase III data determined the optimal second-line therapy (BELINDA, ZUMA-7, and TRANSFORM clinical trials) (17, 29, 30). In 2022, a two-year follow-up retrospective study reported favorable survival following auto-HSCT (26). Long-term follow-ups of CAR-T cell therapy are yet to be performed. In our study, we analyzed survival for as long as two years and found that auto-HSCT was associated with long-term beneficial OS and PFS. Interestingly, it was reported in TRANSFORM that CAR-T cell therapy was superior to standard care, with a longer PFS (30). In the ZUMA-7 study, CAR-T cell therapy demonstrated a clinically meaningful improvement in EFS compared with standard care, which seemed to contradict our results (17). However, according to their studies, only 36% of patients in ZUMA-7 and 47% of patients in TRANSFORM eventually received auto-HSCT after high-dose chemotherapy (30). Over half of the assessable patients failed to undergo auto-HSCT, and we hypothesized that the low transplant rate was responsible for the inferior survival of standard care. No significant difference in survival was observed in BELINDA between CAR-T cell therapy and auto-HSCT, with a transplant rate of 32.5% (29). The best alternative for R/R DLBCL remains controversial, and further studies are warranted to explore the prognosis of patients with different transplant rates. In our study, all patients evaluated in the auto-HSCT group received auto-HSCT and exhibited superior two-year survival compared with the CAR-T group. Age, ECOG performance status, and prior lines of therapy are also factors for efficacy in key CAR-T cell therapy clinical trials (ZUMA-1, JULIET, and TRANSCEND) (45). The median age at diagnosis of DLBCL is in the sixties, and younger patients appear to have more options for treatment, given adequate bone marrow reserve, rapid drug metabolism, limited complications, or suitable physical function (46, 47). Similarly, patients with higher ECOG scores experienced higher mortality and a higher risk of a second relapse. A subgroup analysis of previous therapeutic lines was performed, where there was no difference in OS and PFS between auto-HSCT and CAR-T cell therapy, focusing on those with one or two prior lines of treatment. The auto-HCT group exhibited superior OS in patients with more therapeutic lines. Given the physical condition, adverse events, and treatment cost, patients with more than three median previous therapeutic lines tended to give up CAR-T cell therapy (45). Therefore, we performed stratified analysis by median previous therapies of no more than three lines and found that auto-HSCT was associated with promising long-term survival in each subgroup, which was consistent with overall outcomes. There appears to be a consensus on auto-HSCT for patients who achieve CR. In the CAR-T era, relapsed chemosensitive DLBCL patients not achieving CR following salvage therapy are increasingly receiving CAR-T cell therapy instead of auto-HSCT (48). Considering those with a baseline status of PR, auto-HSCT has the potential to bring about improved OS and a lower incidence of relapse compared to CAR-T cell therapy (26). Hence, we believe that patients should be prudent in choosing CAR-T cell therapy in lieu of auto-HSCT for the following reasons: first, it was still controversial to choose a proper treatment for R/R DLBCL patients to date (49); second, no other options are available in case of treatment failure or relapse after CAR-T cell therapy (50). In addition, the considerable economic burden of CAR-T cell therapy should be considered (51).

Three CAR-T cell products targeting the CD19 antigen on B cells, approved to date, were included in our study. Nevertheless, we were unable to separately analyze the efficacy of different anti-CD19 CAR-T cell infusion products because of the limited number of available clinical trials. Matching-adjusted indirect comparison showed similar ORR, CR, OS, and PFS between liso-cel and axi-cel, while tisa-cel was associated with a superior objective response, CR, and OS to axi-cel (52, 53). As the heterogeneity was acceptable and intergroup outcomes were similar in our study, we did not perform further analysis of CAR-T cell products. Due to the absence of direct head-to-head studies of various anti-CD19 CAR-T cell infusion products, future studies are warranted to further elucidate their relative efficacy.

To the best of our knowledge, this is the first systematic review and meta-analysis to integrate the available published data and compare the therapeutic effects of CAR-T and auto-HSCT in patients with R/R DLBCL. Patients were more likely to achieve a considerable remission rate with CAR-T cell therapy. Those who succeeded in receiving auto-HSCT showed promising long-term survival compared with CAR-T cell therapy. High CAR-T cell peak concentrations in the first 28 days after infusion accounted for a better ORR than auto-HSCT, but the longevity seemed to be worse (54). We were unable to discuss adverse events because of the scarcity of data in the auto-HSCT group. Most patients underwent high-dose chemotherapy before auto-HSCT, and it was difficult to determine whether the adverse events were caused by standard care or by auto-HSCT only. Nevertheless, our study offers some individual suggestions for patients with R/R DLBCL based on their physical condition as well as previous therapies, which are highly clinically relevant. We hope that this review will be thought-provoking and bring attention to this field as more head-to-head clinical trials comparing CAR-T cell therapy and auto-HSCT are needed.

## 5 Conclusion

CAR-T cell therapy and auto-HSCT are crucial treatment strategies for patients with R/R DLBCL. CAR-T cell therapy showed superior ORR and PR compared to auto-HSCT in patients with R/R DLBCL. However, no significant difference in CR was observed between the two groups. Auto-HSCT showed a better long-term OS and PFS than CAR-T cell therapy in patients with R/R DLBCL in different age groups, prior lines of therapy, and ECOG subgroups. In the absence of detailed information on the enrolled patients, we were unable to perform further subgroup analyses and explore additional prognostic factors; therefore, additional RCTs are needed. In any case, individual characteristics should be considered when choosing an appropriate treatment option.

## Data availability statement

The original contributions presented in the study are included in the article/**Supplementary Material**. Further inquiries can be directed to the corresponding author.

## Author contributions

ZZ and LL designed the study. LT, CL and YZ made the statistical plan. LT performed the key analyses. JW, SL, YJ and WW extracted the data. DX, YaL, JG and HX summarized the data. HS and YuL assisted in data interpretation and quality assessment. LT wrote the manuscript. CL revised the manuscript. JS offered constructive suggestions and made major revisions which improved the quality of the manuscript. All authors have read and approved the manuscript. All authors contributed to the article and approved the submitted version.
