# Supplementary material for: Efficacy of chimeric antigen receptor T cell therapy and autologous stem cell transplant in relapsed or refractory diffuse large B-cell lymphoma: A systematic review
Source: Front Immunol. 2023 Jan 17;13:1041177. doi: 10.3389/fimmu.2022.1041177 (PMC9886865; doi:10.3389/fimmu.2022.1041177)
Supplement: Supplementary file 1 [file DataSheet_1.docx]

**Additional file 1 :FigS1** Begg’s test and the Egger’s test a)ORR b)OS c)PFS


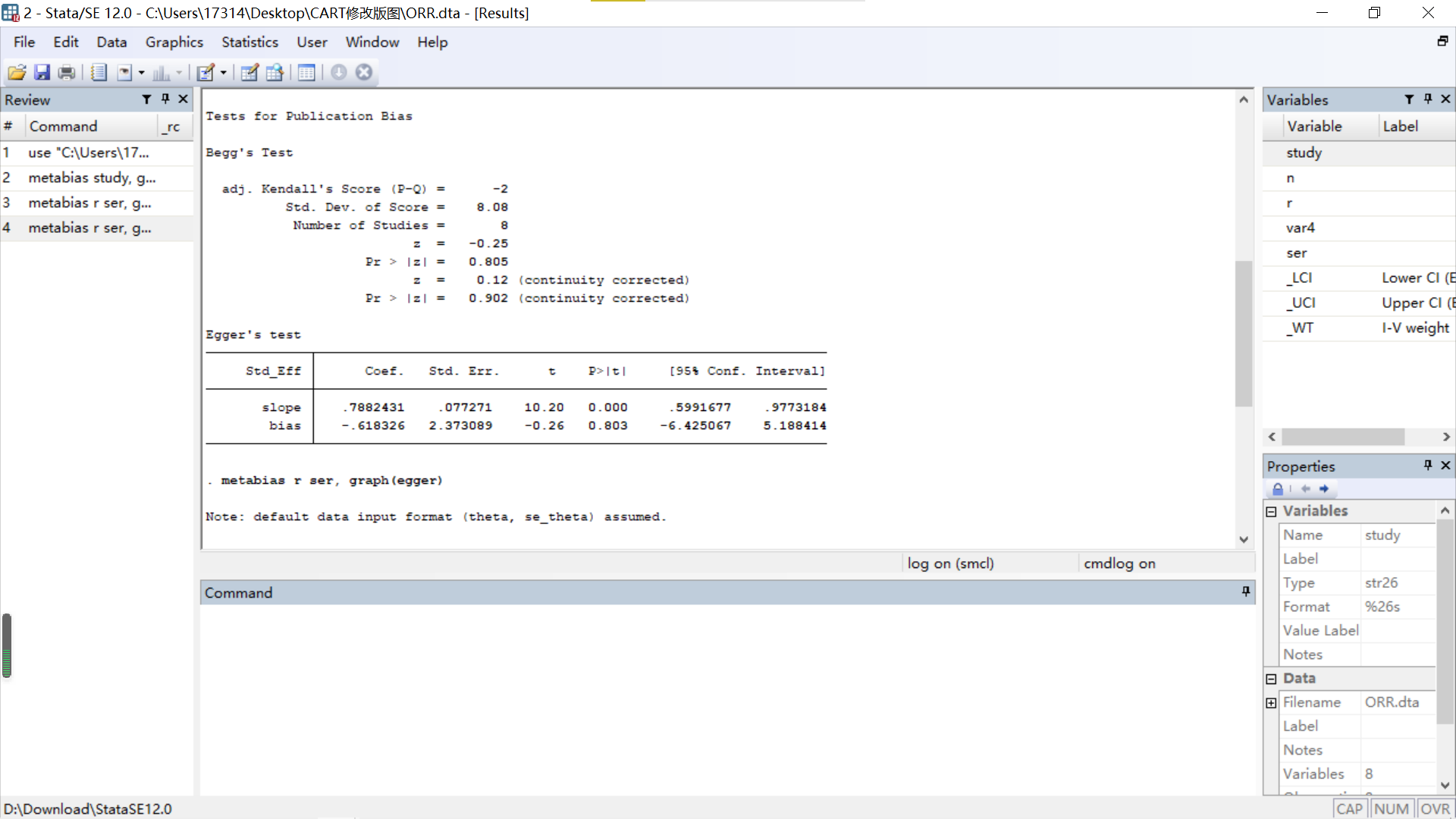


Begg’s test and the Egger’s test a)ORR


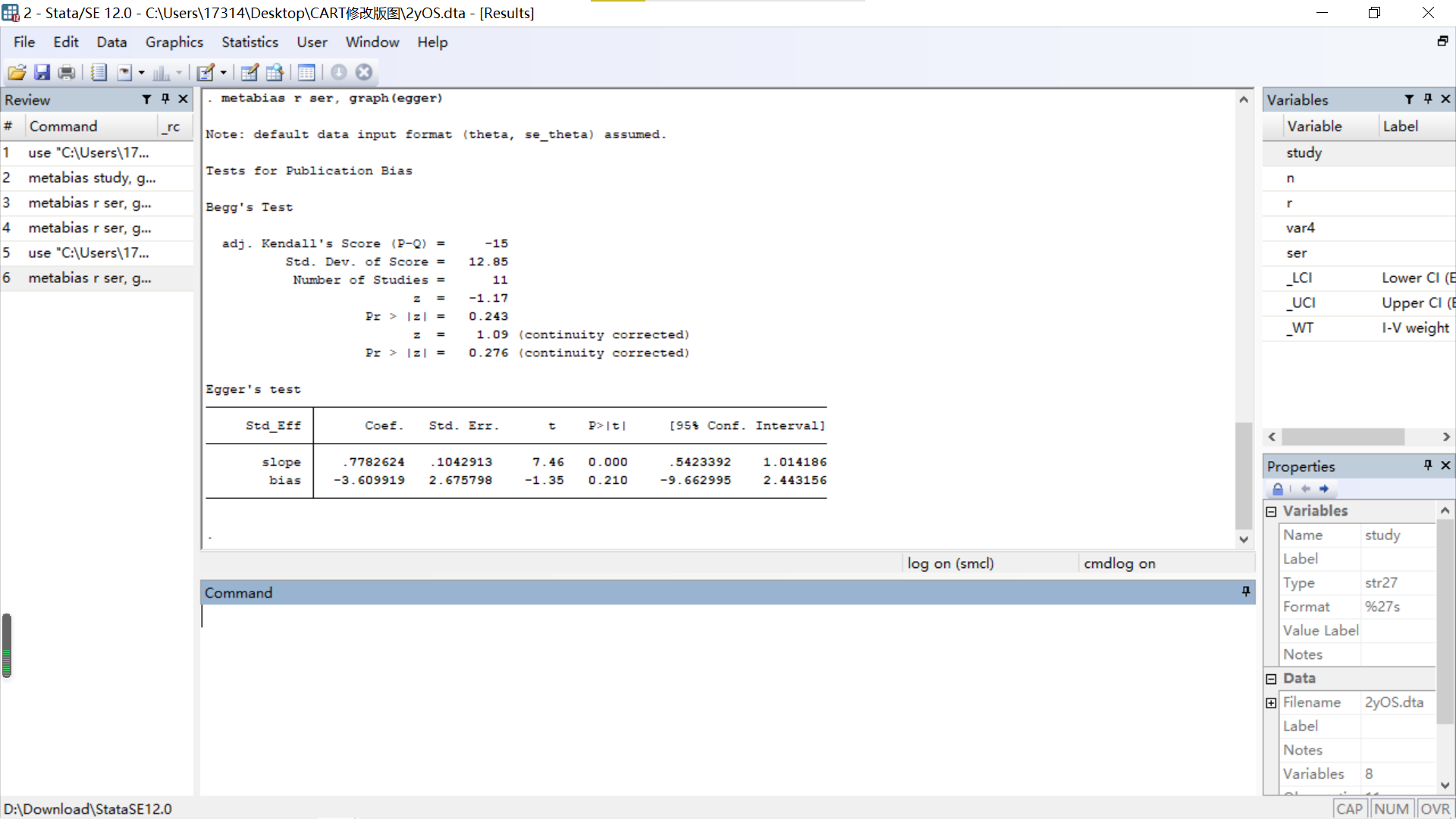


Begg’s test and the Egger’s test b)OS


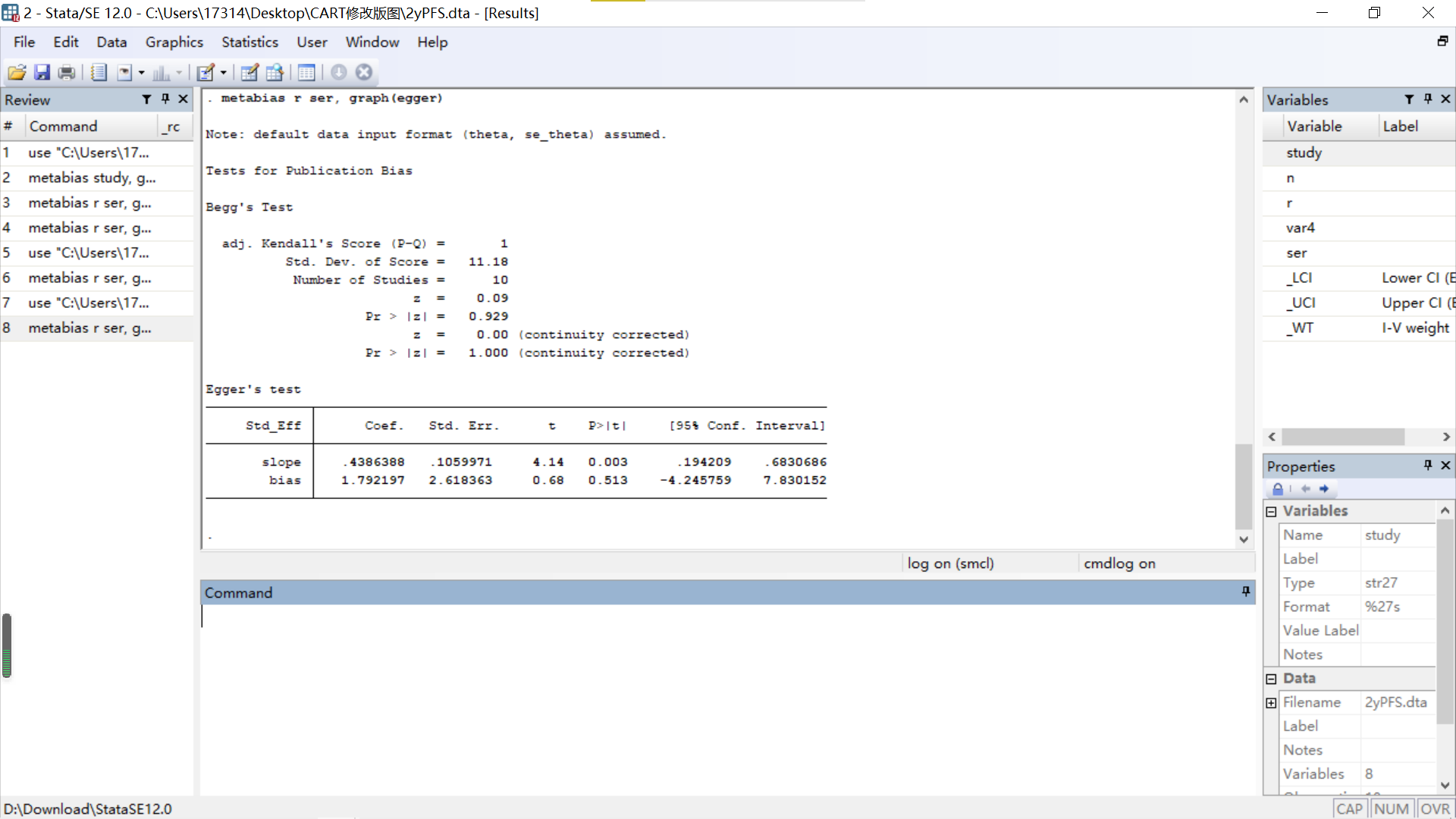


Begg’s test and the Egger’s test c)PFS
